# Metagenomic Diagnosis for a Culture-Negative Sample From a Patient With Severe Pneumonia by Nanopore and Next-Generation Sequencing

**DOI:** 10.3389/fcimb.2020.00182

**Published:** 2020-05-05

**Authors:** Kaiying Wang, Peihan Li, Yanfeng Lin, Hongbin Chen, Lang Yang, Jinhui Li, Tingyan Zhang, Qichao Chen, Zhonghong Li, Xinying Du, Yusen Zhou, Peng Li, Hui Wang, Hongbin Song

**Affiliations:** ^1^College of Military Medicine, Academy of Military Sciences, Beijing, China; ^2^Center for Disease Control and Prevention of PLA, Beijing, China; ^3^State Key Laboratory of Pathogen and Biosecurity, Beijing Institute of Microbiology and Epidemiology, Beijing, China; ^4^Department of Clinical Laboratory, Peking University People's Hospital, Beijing, China; ^5^College of Chemistry and Chemical Engineering, Henan University of Technology, Zhengzhou, China; ^6^College of Environmental and Chemical Engineering, Nanchang Hangkong University, Nanchang, China

**Keywords:** metagenomic next-generation sequencing (mNGS), nanopore sequencing, whole genome sequencing, clinical diagnosis, severe pneumonia

## Abstract

Rapid and accurate etiologic diagnosis accelerates targeted antimicrobial therapy. Metagenomic analysis has played a critical role in pathogen identification. In this study, we leveraged the advantages of both the MinION and BGISEQ-500 platforms to make a bacteriologic diagnosis from a culture-negative lung tissue sample from an immunocompromised patient with severe pneumonia. Real-time nanopore sequencing rapidly identified *Klebsiella pneumoniae* by an 823 bp specific sequence within 1 min. Genomic analysis further identified *bla*_SHV-12_, *bla*_KPC-2_, *bla*_TEM-1_, *bla*_CTX-*M-*65_, and other resistance genes. The same sample was further sequenced on the BGISEQ-500 platform, which presented consistent results regarding the most top dominant pathogens and provided additional information of resistance genes. Revised antibiotic treatment was followed by the patient's clinical recovery. Though sample preparation and the interpretation of final results still need to be improved further, metagenomic sequencing contributes to the accurate diagnosis of culture-negative infections and facilitates the rational antibiotic therapy.

## Introduction

Infectious diseases are major causes of morbidity and mortality (Simner et al., 2017). Diagnostic uncertainty confounds timely and effective therapy, and amplifies the risk of adverse clinical outcomes (Morens and Fauci, 2012). Rapid etiologic diagnosis can facilitate timely and rational antimicrobial treatment (Li et al., 2018). However, the etiologies of 60% clinical infections remain undiagnosed (Simner et al., 2017). Metagenomic next-generation sequencing (mNGS) has emerged with more rapid and accurate diagnostic advantages than traditional methods, especially in culture-negative samples (Greninger et al., 2015; Schmidt et al., 2016; Gong et al., 2018; Li et al., 2018). In addition, genotypic results can identify antibiotic resistance genes and provide markers to facilitate epidemiologic studies (Gwinn et al., 2019).

Currently, different sequencing platforms have been applied to mNGS of clinical samples (Li et al., 2018; Sanderson et al., 2018). MinION (Oxford Nanopore Technologies, Oxford, UK) sequencer shows obvious advantages in clinical utility with its rapid sequencing and real-time analysis (Charalampous et al., 2019). A previous study revealed that pathogens in blood samples could be detected within 10 min by nanopore sequencing (Greninger et al., 2015). However, the sequences generated by MinION still have a high error rate of 5–15% (Rang et al., 2018). It is necessary to combine NGS platforms to improve accuracy.

By combining the advantages of both Nanopore and NGS platforms, metagenomic sequencing can not only achieve a rapid pathogenic diagnosis, even in culture-negative samples, but also maximize genomic information. In this study, we identified *Klebsiella pneumoniae* in a culture-negative lung tissue sample by combining the MinION and BGISEQ-500 sequencing platforms during the treatment of an immunocompromised patient with severe pneumonia. Analysis of antibiotic resistance genes provided valuable data to guide rational antibiotic therapy. These results indicate that metagenomic sequencing has the potential to improve pathogen identification in clinical samples, especially in culture-negative cases.

## Materials and Methods

### Ethics Statement

Application of the study was submitted to the Ethical Review Committee of Peking University People's Hospital (ID: 2019HB134) and supervised by Center for Disease Control and Prevention of PLA. Verbal consents were obtained as no personally-identifiable data were included.

### Patient Information

A 63-year-old man admitted for the evaluation and treatment of chronic lymphocytic leukemia (Rai: stage III, Binet: stage C) developed fever and cough during his hospitalization. Physical findings supported a clinical diagnosis of severe pneumonia. Computerized tomography (CT) showed multiple infiltrates in both lungs. On the 17th hospital day, he underwent computerized tomography (CT)-guided stereotactic lung biopsy. Lung tissue obtained at biopsy was taken to the hospital clinical laboratory for cultures. Additional findings included leukocytosis of 72.7 × 10^9^/L and elevated C-reactive protein (154.95 mg/L). Cultures of blood, respiratory secretions and lung tissue were negative. The infection was refractory to antibiotic therapy. The patient had a fever on the 29th hospital day, which lasted for days and made the condition worse. On the 37th hospital day, lung biopsy tissue slurry was sent to our laboratory for cultures and mNGS.

### Sample Isolation and Identification

Approximately 90 μl of lung biopsy tissue slurry was obtained. A 1 μl aliquot was cultured on Luria-Bertani (LB) agar medium and incubated at 37°C. A 10-μl aliquot was added to 5 ml LB broth medium and incubated in 37°C 180 rpm for 72 h.

Sputum samples from different time points were cultured on chocolate agar, blood agar, and eosin methylene blue agar at 35°C. Sabouraud dextrose agar without chloramphenicol incubated at 35°C was used to isolate fungi. Bacteria were identified by the Vitek2 automated system (BioMérieux, Marcy-l'Etoile, France).

### Antimicrobial Susceptibility Testing

One hundred and forty five microliter bacterial suspension of *P. aeruginosa* with a 0.5-McFarland turbidity was mixed with 3 ml 0.45% NaCl solution. The minimum inhibitory concentrations (MICs) of piperacillin, piperacillin/tazobactam, cefotetan, ceftazidime, cefepime, imipenem, meropenem, amikacin, gentamicin, tobramycin, ciprofloxacin, levofloxacin was determined by the Vitek2 compact system with the AST-GN09 card. The antimicrobial sensitivity results were analyzed according to the Clinical and Laboratory Standards Institute guidelines (CLSI, 2019).

### Metagenomic Sequencing

Genomic DNA from the remaining 75 μl of the lung biopsy tissue slurry was extracted using the High Pure PCR Template Preparation Kit (Cat. No: 11796828001, Roche, Switzerland) following the manufacturer's protocol. Final genomic DNA was eluted with 30 μl elution buffer, and DNA concentration was calculated by Qubit 3.0 (Life Invitrogen, USA). An aliquot of 7.5 μl extracted DNA was used for nanopore library preparation with a Rapid Sequencing Kit (Cat. No: SQK-RAD004, Oxford Nanopore Technologies, UK) according to the manufacturer's protocol. A sample containing 16 ng of genomic DNA was loaded into the Flow Cell (FLO-MIN106 R9 Version) and sequenced on the MinION device using the SQK-RAD004_Base-caller script of the MinKNOW 3.1.19 software. The remaining extracted DNA (44.94 ng genomic DNA) was used for BGISEQ-500 library preparation according to the protocol of MGIEasy DNA Library Prep Kit (MGI, China). DNA was fragmented into 300 bp with micro-TUBE (Cat. No: 520045, Covaris, USA) on Covaris M220. The library size was evaluated with Qsep100 (Taiwan Guangding Biological, China). Forty fmol of DNA were used for nanoball replication according to the manufacturer's protocol (BGISEQ-500RS High-throughput Sequencing Set PE100 V3.0) and sequenced on the BGISEQ-500 platform.

### Whole Genome Sequencing of *Pseudomonas aeruginosa*

Genomic DNA of cultured *P. aeruginosa* was extracted as described as above. One hundred and twenty nanogram genomic DNA was fragmented into 450 bp with micro-TUBE (Cat. No: 520045, Covaris, USA) on Covaris M220 and used for library preparation using NEBNext® Ultra™ II DNA Library Prep Kit (Cat. No: E7645S, NEB, USA) according to the manufacturer's manual. Short-read sequencing was performed on the Illumina MiSeq platform with an insert of 450 bp. Library for Nanopore sequencing was directly prepared with 400 ng genomic DNA according to the manufacturer's protocol of Rapid Sequencing Kit (Cat. No: SQK-RAD004, Oxford Nanopore Technologies, UK) and sequenced on the MinION platform with a R9.4.1 Flow Cell.

### Bioinformatics Analysis

Raw FAST5 files from MinION were base-called using the Basecalling v1.14 pipeline, and PycoQC v2.2.4 (Leger and Leonardi, 2019) was used for quality control. SOAPnuke v2.0.7 (Chen et al., 2017) was used to remove low-quality reads generated by BGISEQ-500. The mNGS data from two platforms were classified using Centrifuge v1.0.4 with–min-hitlen 60 (Kim et al., 2016). *K. pneumoniae* HS11286 (NC_016845.1) was used as the reference genome. Genomic sequences of *K. pneumoniae* from mNGS were mapped to the reference using BWA v0.7.12 (Li and Durbin, 2009) and Samtools v1.3 (Wysoker et al., 2009). Capsular serotype and virulence factors of *K. pneumoniae* were identified using Kaptive (Wick et al., 2018) and reference virulence genes provided by the Pasteur Institute (http://bigsdb.web.pasteur.fr/klebsiella/klebsiella.html), respectively. Seven housekeeping genes of *K. pneumoniae* (*gapA-infB-mdh-pgi-phoE-rpoB-tonB*) and *P. aeruginosa* (*acsA-aroE-guaA-mutL-nuoD-ppsA-trpE*) were used for typing through the multi-locus sequence typing (MLST) web server (Larsen et al., 2012). Whole genome of *P. aeruginosa* were assembled *de novo* using MECAT (Xiao et al., 2017) and re-quantified by mapping the MiSeq data to the assembly. Phylogenetic maximum likelihood tree of two *P. aeruginosa* strains with other 29 available *P. aeruginosa* genomes was constructed based on the concatenated SNPs using CSI phylogeny 1.4 (Kaas et al., 2014). Antibiotic-resistant genes were identified by BLAST against the Comprehensive Antibiotic Resistance Database (CARD) (Jia et al., 2016).

## Result

### Pathogen Isolation and Identification

**Figure 1:**
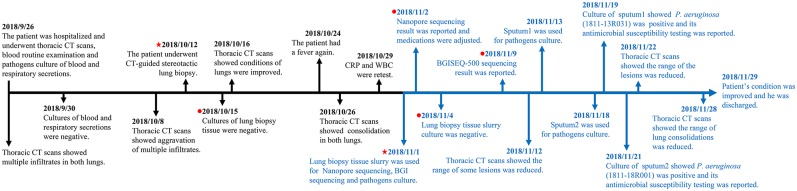
Timeline from hospitalization to patient discharge. The markers in black show the events from hospitalization to pre sequencing and the markers in blue show the events from sequencing to patient discharged. The red pentagram indicates the dates of taking samples and sequencing, and the red dot indicates the dates of culturing and sequencing results, respectively.

Cultures of lung biopsy tissue slurry were negative in both the hospital clinical laboratory and our laboratory. Two isolates were recovered by cultures of sputum samples submitted later in the patient's hospital course. Both isolates were identified as *P. aeruginosa* (no. 1811-13R031 and no. 1811-18R001) (Figure 1). No fungi were recovered.

### Pathogen Detection and Resistance Analysis by Metagenomic Sequencing

**Figure 2:**
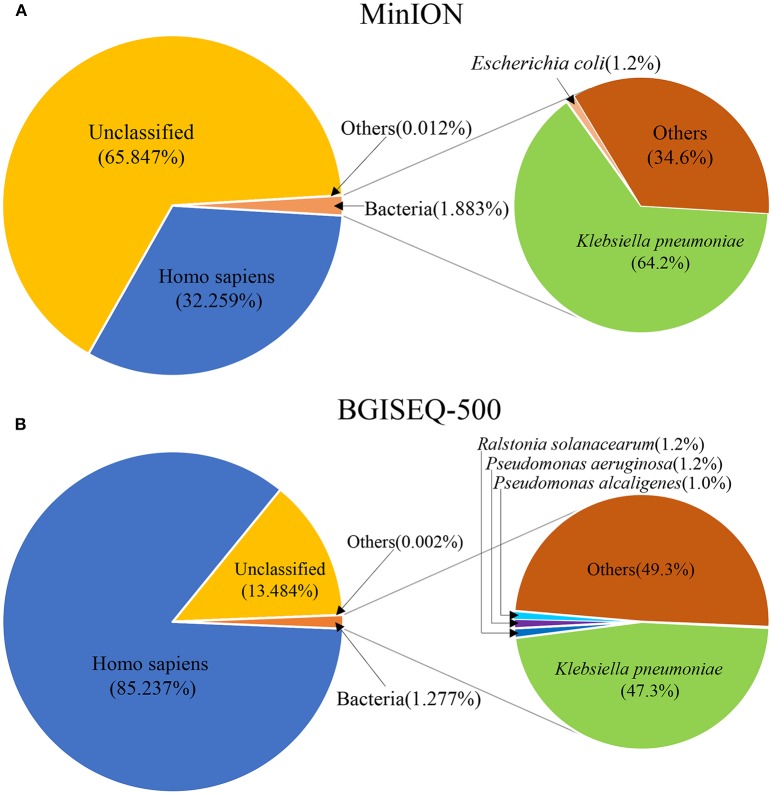
The species composition of mNGS data from MinION **(A)** and BGISEQ-500 **(B)**. “Others” represents the sum of all species which had a proportion <1%.

DNA from the lung biopsy slurry was extracted in ~30 min. Only 16.05 ng of DNA was used for nanopore sequencing, which generated 79.23 MB data with a total of 34,831 reads in 12.14 h. The percentage of *Homo sapiens* reads, unclassified reads and different microbial reads of MinION data are shown in Figure 2A. Bacterial sequences accounted for 1.883% (656 sequences) in all sequences. The *K. pneumoniae* specific sequences accounted for 64.2% (421 sequences) of the bacterial sequences (Table 1). The first specific 823 bp read of *K. pneumoniae* was detected within 1 min (Figure S1). The 421 sequences of *K. pneumoniae* were detected within 495 min. Genomic coverage of *K. pneumoniae* reached 45% by MinION (Figure S2). Moreover, the remaining 235 sequences matched other bacteria.

**Table 1:** Top ten species of MinION- and BGISEQ-500-based mNGS.

**Number**	**MinION**		**BGISEQ-500**	
	**Pathogen**	**No. unique reads**	**Pathogen**	**No. unique reads**
1	Klebsiella pneumoniae^*^	421	Klebsiella pneumoniae^*^	781943
2	Escherichia coli^*^	8	Ralstonia solanacearum^*^	20105
3	Enterobacter cloacae^*^	3	*Pseudomonas aeruginosa*	19578
4	Ralstonia solanacearum^*^	2	*Pseudomonas alcaligenes*	16361
5	*Klebsiella variicola*	2	Klebsiella oxytoca^*^	5670
6	Acinetobacter baumannii^*^	1	Enterobacter cloacae^*^	4439
7	*Citrobacter koseri*	1	*Pseudomonas putida*	3322
8	Klebsiella oxytoca^*^	1	Escherichia coli^*^	2908
9	*Klebsiella quasivariicola*	1	Acinetobacter baumannii^*^	2205
10	*Salmonella enterica*	1	*Delftia tsuruhatensis*	911

* *indicates the species is present on both platforms*.

An aliquot containing 44.94 ng of DNA was used for library construction on the BGISEQ-500 platform, generating 24.5 GB data with a total of 129,512,318 reads. The percentage of *Homo sapiens* reads, unclassified reads, and other microbial reads of BGISEQ-500 data are shown in Figure 2B. There were 1,654,304 reads (1.277%) aligned to bacteria. The *K. pneumoniae* sequences accounted for 47.3% (781,943 sequences) of the bacterial sequences (Table 1). The coverage of the reference genome of *K. pneumoniae* reached 100% (Figure S2). The sequence type and serotype of *K. pneumoniae* belongs to ST11 and K47, respectively. Analysis of 18 virulence genes (*fyuA, irp1, irp2, ybtA, ybtE, ybtP, ybtQ, ybtT, ybtU, ybtX, mrkA, mrkB, mrkC, mrkD, mrkF, mrkH, mrkI, mrkJ*) indicated that the *K. pneumoniae* did not belong to a hypervirulent strain.

In addition, other bacteria were detected, the sequences number of the top 10 species are shown in Table 1. The top 10 detected species were different between the two platforms, although six of which were consistent (Table 1). There were 19,778 *P. aeruginosa* reads, 16,361 *P. alcaligenes* reads, and 3,322 *P. putida* reads in the BGISEQ-500 data, but no reads of these species were detected in the MinION data. Genome coverage of the *P. aeruginosa* from BGISEQ-500 data was 3.02% using strain 1811-18R001 as reference with the average coverage depth of 0.19 folds.

The identified resistance genes were present in both metagenomic databases (Table 2). Resistance genes *bla*_SHV-12_, *aac(3)-IIa* and *bla*_KPC-2_ were detected by the MinION platform at 29th, 38th, and 56th min of sequencing, respectively. Our results showed that the subtypes of resistance genes identified by the BGISEQ-500 platform have been verified in NCBI with a matching degree of 100%. We detected the following resistance genes: *aadA1, catA1, dfrA1, sul1, aph(3*′*)-Ia, aac(3)-IIa, bla*_SHV-12_, *bla*_KPC-2_, *bla*_TEM-1_, *bla*_CTX-*M-*65_, *fosA, acrA*, and *rmtB*. Among them, *aadA1, catA1, dfrA1, sul1, aph(3*′*)-Ia*, and *aac(3)-IIa* also had high identity on the MinION platform. A 450-bp span at the end of the ~2.6 kb sequence from MinION matches the *bla*_KPC_ gene and the plasmid *pKPC-167* (GenBank ID:MH236906, unpublished). In addition, a 349-bp sequence directly matches the *bla*_SHV-12_ gene. In this sequence, the part 204–341 bp can be matches the 372–516 bp of the *bla*_SHV-12_ gene with an identity of 93.97%.

**Table 2:** Resistance genes detected by the MinION and BGISEQ-500 platforms.

**Classification**	**MinION**		**BGISEQ-500**	
	**Resistance gene**	**Identity**	**Resistance gene**	**Identity**
Aminoglycoside	*aadA1*	88.49%	*aadA1*	100%
	*aac(3)-IIa*	86.67%	*aac(3)-IIa*	100%
	*aph(3′)-Ia*	90.06%	*aph(3′)-Ia*	100%
	–	–	*rmtB*	100%
Sulfonamide	*sul1*	89.58%	*sul1*	100%
Trimethoprim	*dfrA1*	90.00%	*dfrA1*	100%
Beta-lactam	*bla*_SHV-12_	segment	*bla*_SHV-12_	100%
	*bla*_KPC-2_	segment	*bla*_KPC-2_	100%
	–	–	*bla*_TEM-1_	100%
			*bla*_CTX-*M-*65_	100%
Chloramphenicol	*catA1*	92.61%	*catA1*	100%
Fosfomycin	–	–	*fosA*	100%
Quinolones or chloramphenicol	–	–	*acrA*	100%

–*, no gene for this kind of drug*.

### Genomic Analysis of Two *Pseudomonas aeruginosa* Isolates

Analysis of MinION and Miseq data revealed no differences between the genomes of the two *P. aeruginosa* isolates 1811-13R031 and 1811-18R001. The two *P. aeruginosa* strains (no. 1811-13R031 and no. 1811-18R001) belonged to ST395 and were closely clustered in the same branch of the tree (Figure S3), indicating the two can be referred to as a clone. The genomic sequences of *P. aeruginosa* isolates 1811-13R031 and 1811-18R001 were stored in GenBank with identification codes CP046061 and CP046060, respectively. We also performed genomic drug resistance analysis and standard *in vitro* antimicrobial susceptibility testing of the two *P. aeruginosa* isolates (Tables S1, S2). The 39 resistance genes of the two *P. aeruginosa* isolates were identical. The resistance genes in our *P. aeruginosa* isolates and the metagenome are completely different. Antimicrobial susceptibility testing showed that both *P. aeruginosa* isolates were resistant to cefotetan, imipenem, and meropenem, but were sensitive to amikacin, gentamicin, tobramycin and ciprofloxacin. Interestingly, compared with *P. aeruginosa* 1811-13R031, the second *P. aeruginosa* isolate 1811-18R001 had enhanced resistance to piperacillin, piperacillin/tazobactam, and cefepime (Table S2).

### Rational Therapy Guided by Sequencing

Radiographic findings combined with sequencing results indicated that the patient's pulmonary pathogens were more likely gram-negative bacterial than fungal on the 38th hospital day (Nov. 2nd) (Figure 1). The patient's therapeutic regimen was adjusted to meropenem, tigecycline, caspofungin, posaconazole, voriconazole, and amikacin subsequently. BGISEQ-500 sequencing indicated both *K. pneumoniae* and *P. aeruginosa* may be responsible for the infection on the 45th day (Nov. 9th). Ceftazidime was added for infection treatment. Later, piperacillin and piperacillin/tazobactam were prescribed according to the antimicrobial susceptibility result of *P. aeruginosa* strain 1811-13R031 (Nov. 19th). Considering that there were still few fungal sequences detected by both platforms, antifungal therapy was retained with caspofungin and voriconazole (Table S3).

The newly prescribed antibiotics (tigecycline, amikacin, and ceftazidime) increased the antimicrobial activity of the patient's regimen against *K. pneumoniae* and *P. aeruginosa*. In addition, the side effects of vancomycin, linezolid, moxifloxacin, and sulfamethoxazole were avoided. The patient's condition was improved, and he was discharged after the revision of his treatment.

## Discussion

The predominant pathogen detected by both platforms was *K. pneumoniae*. However, no *K. pneumoniae* was isolated in standard cultures of the lung biopsy tissue. The most likely reason for the detection but not isolation of *K. pneumoniae* is that the sensitivity of standard cultures was reduced by antibiotic therapy given before the lung biopsy tissue was obtained. Sequencing results also revealed the low copy number of *K. pneumoniae* in the sample. However, genomic DNA of *K. pneumoniae* remains and could be detected by high-throughput sequencing. It took ~1 min from beginning of sequencing to obtain the first specific reads of *K. pneumoniae* by MinION. In addition, resistance genes including *bla*_SHV-12_, *aac(3)-IIa* and *bla*_KPC-2_ were detected within one and a half hours. The turn-around time is far more rapid than those of standard cultures and antibiotic sensitivity testing. *K. pneumoniae* DNA was present in the sample, while the number of viable bacteria was below the threshold of detection by standard culture methods. The top 10 species of bacteria detected by the two platforms were different (Table 1). There are 19,578 sequences of *P. aeruginosa* detected by the BGISEQ-500 platform, and the genome coverage is only 3.02%. However, no *P. aeruginosa* sequences were detected by MinION. The amount of DNA, the method of library construction, and the final data output may be explained these different results. Another explanation could be that *K. pneumoniae* was the etiology of the deep pulmonary parenchymal lesions, while *P. aeruginosa* was limited to the upper respiratory tract. Moreover, both platforms displayed reads of *R. solanacearum*, a common plant pathogen that has never been reported to cause human disease. We suspect that the *R. solanacearum* reads were due to contamination.

The distinction between pathogens, commensal microflora, and contaminants requires the clinician to comprehensively assess the patient's clinical status and sequencing results. mNGS was performed 20 days after specimen collection and only a total of 60.1 ng genomic DNA was obtained for subsequent library preparation. However, both platforms yielded enough data to identify the possible responsible pathogen. BGISEQ-500 also detected *P. aeruginosa* a few days ahead of sputum culture. Nanopore sequencing cost 12.5 h, while BGISEQ-500 took over 200 h. Our study further proved the significance of real-time sequencing on clinical diagnostics and indicated that it's worth spending more time to perform high-output sequencing, which could produce abundant data and detect possible pathogens in chronic infections in advance. In the future, optimization of sample acquisition, sample preparation and bioinformatic analysis will be essential to the future application of mNGS technologies in clinical diagnosis.

Antimicrobial resistance genes in clinical samples can be identified from metagenomic data (Grumaz et al., 2016). We found multiple types of resistance genes in our metagenomic sample (Table 2). However, it is difficult to determine which resistance genes were derived from *K. pneumoniae* or from other microorganisms. The challenge of determining the specific reservoirs of resistance genes in metagenomic samples remains to be solved.

*P. aeruginosa* has a variety of active efflux systems that are important causes of intrinsic and acquired multi-drug resistance (Llanes et al., 2004; Pan et al., 2016). In our study, the two *P. aeruginosa* isolates had completely consistent genomes and identical resistance genes. However, *P. aeruginosa* isolate 1811-18R001 showed enhanced *in vitro* resistance to piperacillin, piperacillin/tazobactam and cefepime compared to 1811-13R031. These antibiotics were used continuously during the separation interval between these two isolates. This time interval may have allowed increased expression of efflux systems and to differences in phenotypic drug resistance. Further analysis is needed to understand the mechanisms of different resistance phenotypes.

In conclusion, our study highlights the feasibility of real-time metagenomic sequencing to identify pathogens in culture-negative samples, which may contribute to the accurate diagnosis of clinical infections and facilitate the rational antibiotic therapy.

## Data Availability Statement

The datasets generated for this study can be found in the https://www.ncbi.nlm.nih.gov/nuccore/CP046061.1/; https://www.ncbi.nlm.nih.gov/nuccore/CP046060.

## Ethics Statement

The ethics of the study was reviewed and supervised by Center for Disease Control and Prevention of PLA and Peking University People's Hospital. Verbal consents were obtained as samples were collected through normal surveillance and no personally-identifiable data were included.

## Author Contributions

KW, PHL, and YL performed metagenomic analysis and experiment. HC collected samples. KW wrote the first draft of the manuscript. LY, JL, TZ, QC, ZL, XD, and YZ contributed to manuscript revision. PL, HW, and HS designed the study and revised the manuscript. All authors contributed to manuscript revision, read and approved the submitted version.

## Conflict of Interest

The authors declare that the research was conducted in the absence of any commercial or financial relationships that could be construed as a potential conflict of interest.
