# Supplementary figures and images for: Metagenomic Diagnosis for a Culture-Negative Sample From a Patient With Severe Pneumonia by Nanopore and Next-Generation Sequencing
Source: Front Cell Infect Microbiol. 2020 May 5;10:182. doi: 10.3389/fcimb.2020.00182 (PMC7214676; doi:10.3389/fcimb.2020.00182)

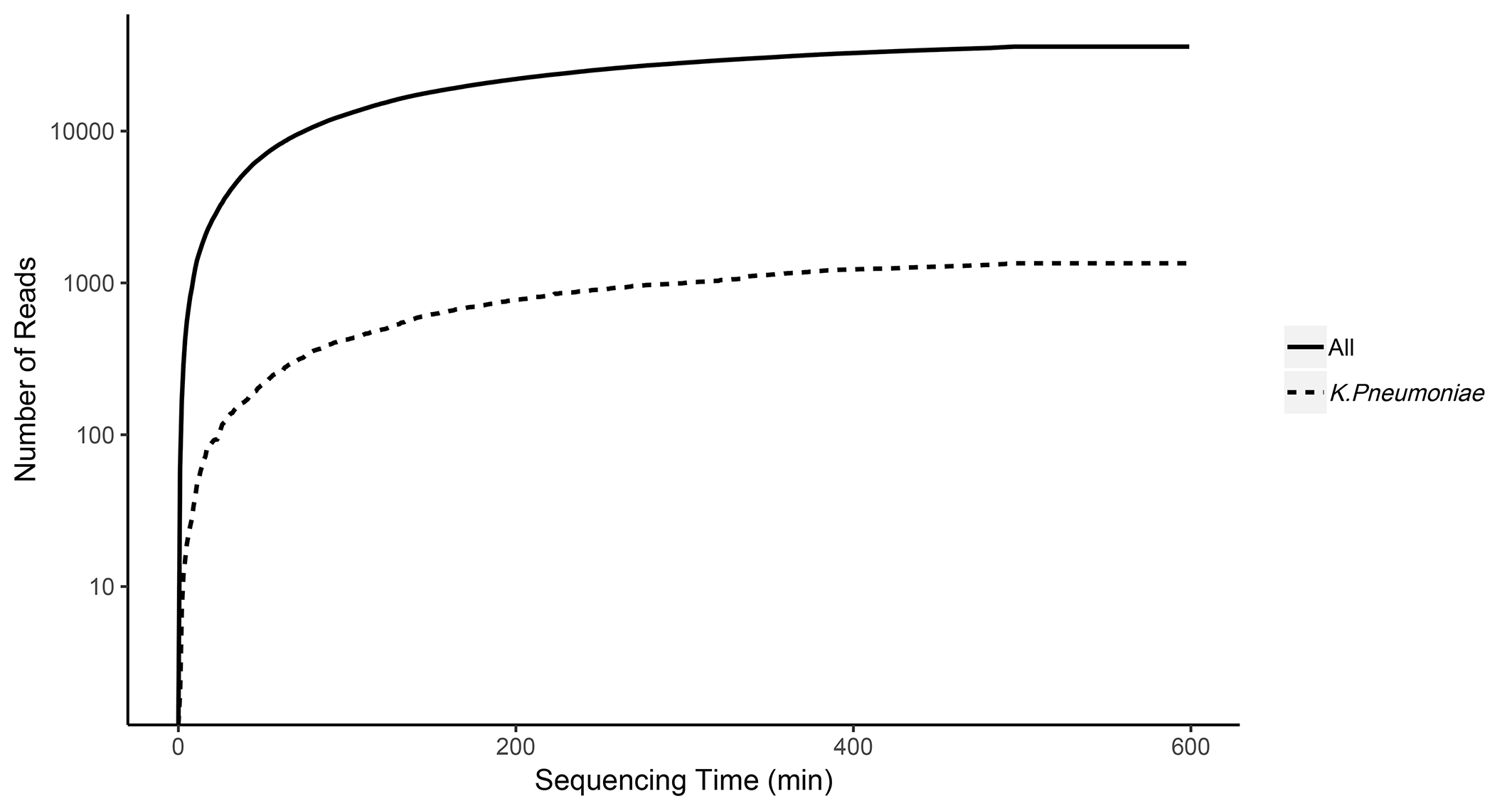

Supplement: Supplementary file 1 [file Image_1.tif]

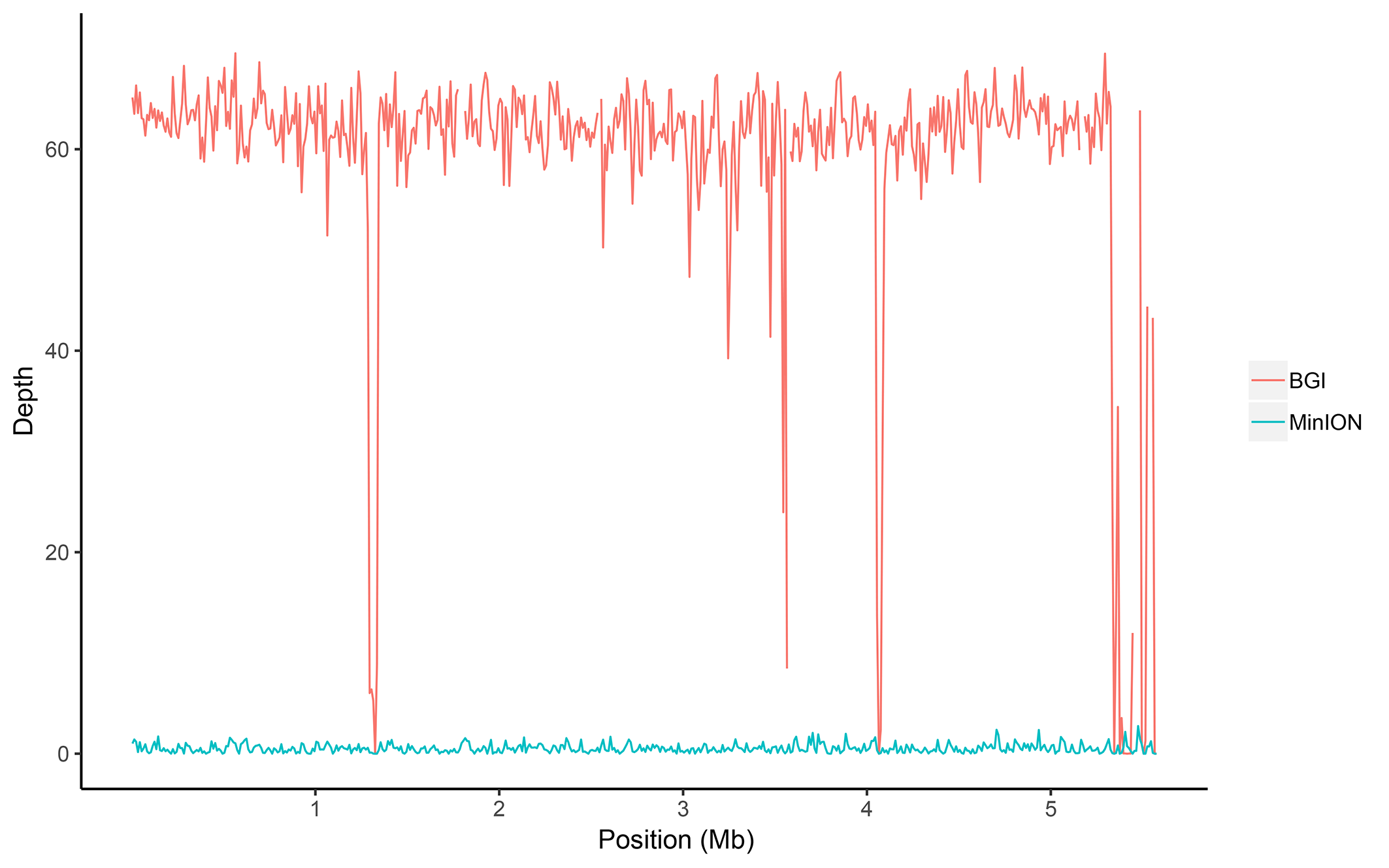

Supplement: Supplementary file 2 [file Image_2.tif]

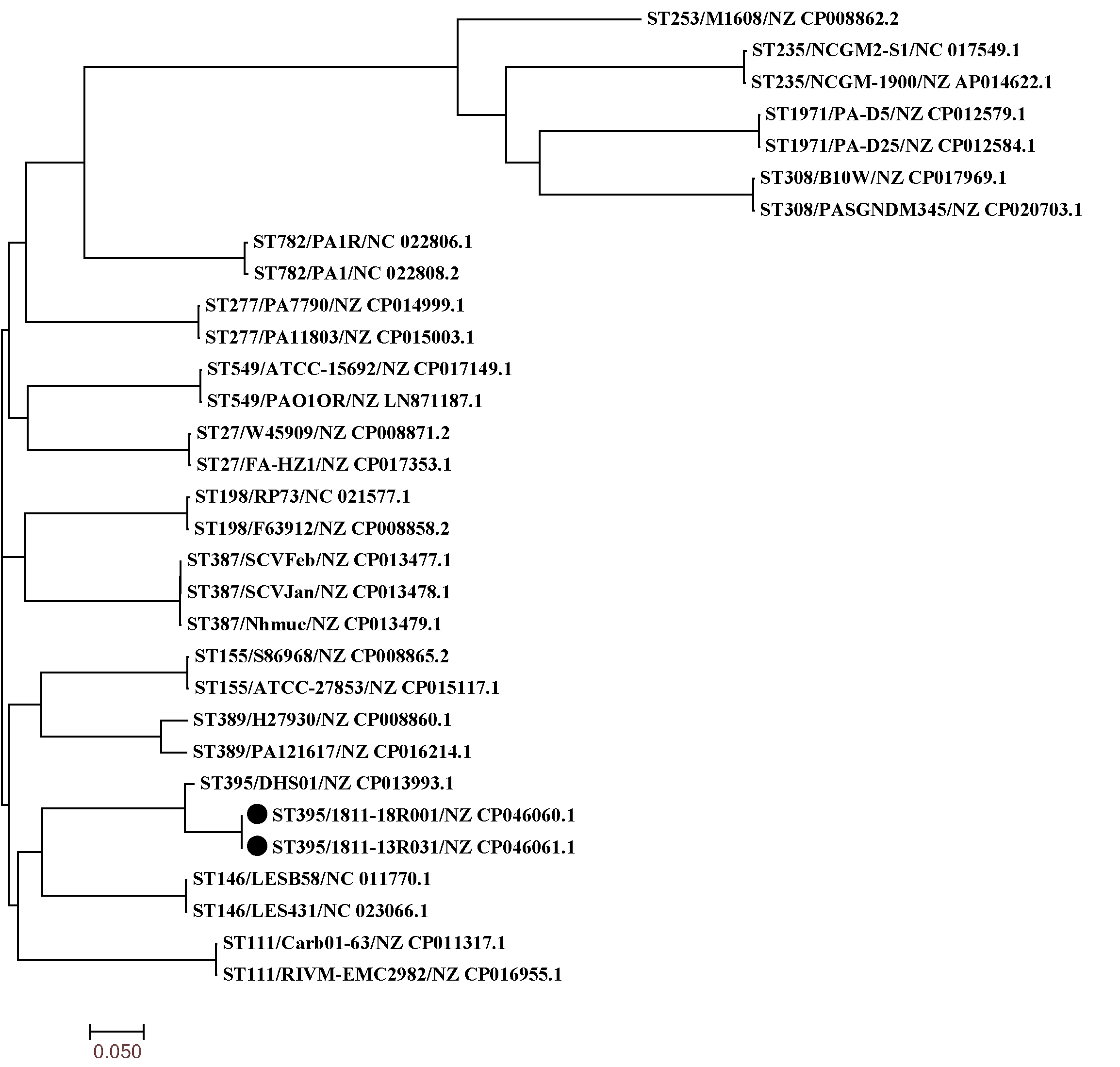

Supplement: Supplementary file 3 [file Image_3.TIF]
