# Supplementary material for: Metagenomic Diagnosis for a Culture-Negative Sample From a Patient With Severe Pneumonia by Nanopore and Next-Generation Sequencing
Source: Front Cell Infect Microbiol. 2020 May 5;10:182. doi: 10.3389/fcimb.2020.00182 (PMC7214676; doi:10.3389/fcimb.2020.00182)
Supplement: Supplementary file 4 [file Data_Sheet_1.docx]

**Supplementary Figure Legends**

**Figure S1** Sequencing time of *K. pneumoniae* detection using MinION. The solid line and the dotted line indicate the number of all sequences and *K. pneumoniae*-specific sequences obtained over time, respectively.

**Figure S2** The genome coverage of *K. pneumoniae* from mNGS data. The red line indicated data from the BGISEQ-500 platform and the light green line indicated data from the MinION platform.

**Figure S3** Phylogenetic maximum likelihood tree of 1811-18R001 and 1811-13R031 with other 29 available *P. aeruginosa* whole genomes from GenBank. Strain 1811-18R001 and 1811-13R031 are marked with black circles.

**Supplementary Tables**

**Table S1** Resistance genes of two *P. aeruginosa* isolates

| 1811-13R031 | 1811-18R001 |
| --- | --- |
| *amrA*, *amrB, aph(3')-IIb* | *amrA, amrB, aph(3')-IIb* |
| *bcr-1* | *bcr-1* |
| *catB7* | *catB7* |
| *mexA, mexB, mexC, mexD, mexE, mexF, mexG, mexH, mexI, mexJ, mexK, mexL, mexM, mexN, mexP, mexQ, mexS, mexV, mexW, mexX, mexY, nalD, pdc-8, oxa-50* | *mexA, mexB, mexC, mexD, mexE, mexF, mexG, mexH, mexI, mexJ, mexK, mexL, mexM, mexN, mexP, mexQ, mexS, mexV, mexW, mexX, mexY, nalD, pdc-8, oxa-50* |
| *nfxB* | *nfxB* |
| *opmD, opmE, opmH* | *opmD, opmE, opmH* |
| *oprJ, oprM, oprN* | *oprJ, oprM, oprN* |
| *triA, triB, triC* | *triA, triB, triC* |

**Table S2** Antibiotic susceptibilities of two *P. aeruginosa* isolates

| Antibiotic | MIC (μg/ml) | |
| --- | --- | --- |
|  | 1811-13R031 | 1811-18R001 |
| Piperacillin | 16/S | 32/I |
| Piperacillin/Tazobactam | 8/S | 64/I |
| Cefotetan | ≥64/R | ≥64/R |
| Ceftazidime | 4/S | 4/S |
| Cefepime | 4/S | 8/S |
| Imipenem | ≥16/R | ≥16/R |
| Meropenem | ≥16/R | ≥16/R |
| Amikacin | ≤2/S | ≤2/S |
| Gentamicin | ≤1/S | ≤1/S |
| Tobramycin | ≤1/S | ≤1/S |
| Ciprofloxacin | ≤0.25/S | ≤0.25/S |
| Levofloxacin | 1/S | 1/S |

**Table S3** Pre- and post- sequencing medication of the therapeutic regimen

| Medication | Pre-sequencing medication | Post-sequencing medication | | | |
| --- | --- | --- | --- | --- | --- |
|  | From first to 37th hospital day (Nov. 1st) | First adjustment  (Nov. 2nd) | Second adjustment  (Nov. 5-6th) | Third adjustment  (Nov. 9th) | Fourth adjustment  (Nov. 19th) |
| Antibiotics | Imipenem/cilastatin | Meropenem | Meropenem | Ceftazidime | Piperacillin |
|  | Cefoperazone/sulbactam | Tigecycline | Tigecycline | ─ | Piperacillin/tazobactam |
|  | Vancomycin | ─ | Amikacin | ─ | ─ |
|  | Moxifloxacin | ─ | ─ | ─ | ─ |
|  | Linezolid | ─ | ─ | ─ | ─ |
|  | Meropenem | ─ | ─ | ─ | ─ |
|  | Sulfamethoxazole | ─ | ─ | ─ | ─ |
|  | Piperacillin | ─ | ─ | ─ | ─ |
|  | Piperacillin/tazobactam | ─ | ─ | ─ | ─ |
| Antifungals | Voriconazole | Posaconazole | Voriconazole | Voriconazole | Caspofungin |
|  | Amphotericin B | Caspofungin | Caspofungin | Caspofungin | ─ |
|  | Caspofungin | ─ | ─ | ─ | ─ |

Note: The drugs in the table were arranged according to the order of use.
